# Discovery of Jogalong virus, a novel hepacivirus identified in a *Culex annulirostris* (Skuse) mosquito from the Kimberley region of Western Australia

**DOI:** 10.1371/journal.pone.0227114

**Published:** 2020-01-03

**Authors:** Simon H. Williams, Avram Levy, Rachel A. Yates, Nilusha Somaweera, Peter J. Neville, Jay Nicholson, Michael D. A. Lindsay, John S. Mackenzie, Komal Jain, Allison Imrie, David W. Smith, W. Ian Lipkin

**Affiliations:** 1 Center for Infection and Immunity, Mailman School of Public Health of Columbia University, New York, New York, United States of America; 2 Faculty of Health and Medical Sciences, University of Western Australia, Nedlands, Western Australia, Australia; 3 PathWest Laboratory Medicine WA, Nedlands, Western Australia, Australia; 4 Environmental Health Directorate, Public and Aboriginal Health Division, Department of Health, Western Australia, Perth, Western Australia, Australia; 5 Faculty of Health Sciences, Curtin University, Perth, Western Australia, Australia; Keele University Faculty of Natural Sciences, UNITED KINGDOM

## Abstract

The discovery of hepaciviruses in non-human hosts has accelerated following the advancement of high-throughput sequencing technology. Hepaciviruses have now been described in reptiles, fish, birds, and an extensive array of mammals. Using metagenomic sequencing on pooled samples of field-collected *Culex annulirostris* mosquitoes, we discovered a divergent hepacivirus-like sequence, named Jogalong virus, from the Kimberley region in northern Western Australia. Using PCR, we screened the same 300 individual mosquitoes and found just a single positive sample (1/300, 0.33%). Phylogenetic analysis of the hepacivirus NS5B protein places Jogalong virus within the genus *Hepacivirus* but on a distinct and deeply rooted monophyletic branch shared with duck hepacivirus, suggesting a notably different evolutionary history. Vertebrate barcoding PCR targeting two mitochondrial genes, cytochrome *c* oxidase subunit I and cytochrome *b*, indicated that the Jogalong virus-positive mosquito had recently fed on the tawny frogmouth (*Podargus strigoides*), although it is currently unknown whether this bird species contributes to the natural ecology of this virus.

## Introduction

Hepaciviruses are positive-sense RNA viruses in the family *Flaviviridae*. Hepaciviruses are difficult to culture; thus, their diversity was underappreciated until the advent of high throughput sequencing (HTS). The genus *Hepacivirus* comprises at least fourteen species that infect humans [1], and other mammals including rodents [2–6], cows [7, 8], horses [9], primates [10, 11], and bats [12]. A survey of Australian ticks also identified a hepacivirus from an *Ixodes holocyclus* tick that fed on a long-nosed bandicoot [13]. Metagenomic analyses of fish and reptiles uncovered the first non-mammalian hepaciviruses [14, 15]. A recent study investigating the etiology of severe disease in ducks identified a highly prevalent and divergent hepaci-like viral sequence in 70% of ducks collected over a wide geographical area [16]. Aside from two turtle hepaciviruses that share a common ancestor with the rodent Hepacivirus J (*Myodes gareolus*), the remaining non-mammalian viruses represent a diverse and separate clade of hepaciviruses. Despite the remarkable evolutionary distance separating these hosts, hepaciviruses have maintained an affinity for liver infection [14].

Here, we describe the discovery of Jogalong virus (JgV) from a single *Culex annulirostris* mosquito from the Kimberley region of Western Australia. The unexpected discovery of a hepacivirus sequence in an invertebrate raised suspicion that JgV may represent partially digested material from a blood meal. Subsequent vertebrate barcoding PCRs suggest that the true host may be of avian origin.

## Methods

### Mosquito collection

**Fig 1:**
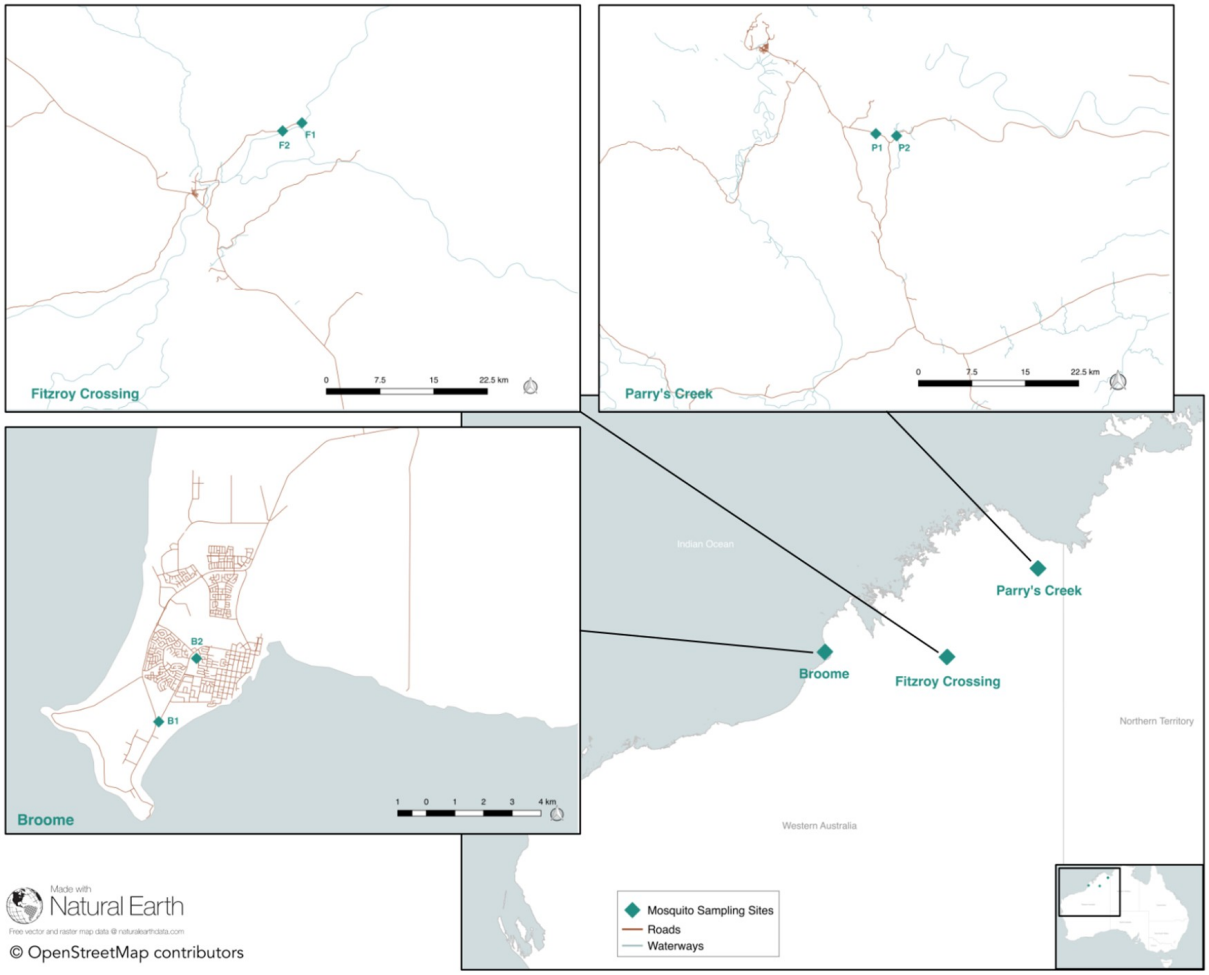
Map of northern Western Australia. Mosquito trap locations for three sites are marked with a green triangle. The inset maps indicate the locations of each of two traps located at each site. Map prepared using QGIS v2.18.15 (http://qgis.osgeo.org), OpenStreetMap, Natural Earth, and Mainroads Western Australia (https://portal-mainroads.opendata.arcgis.com/). The baselayer shapefile was obtained from the Australian Government data portal (https://www.data.gov.au/).

We trapped adult mosquitoes using Encephalitis Virus Surveillance CO_2_-baited traps [17]. Mosquitoes were collected from three sites located in the Kimberley region of Western Australia during March and April 2018 as part of routine arboviral surveillance [18]. Mosquitoes trapped from the townships of Broome and Fitzroy Crossing were collected from Public land, while mosquitoes collected from the rotunda within Geikie Gorge National Park (Fitzroy Crossing) and from Parry Lagoons Nature Reserve (Parry’s Creek) were collected under Department of Parks and Wildlife, Western Australia, Permit number (08-001839-1) (Fig 1). At each site, two traps were located approximately 2.5 km apart. Mosquitoes were separated by species using morphologic criteria [19], and 50 *Cx*. *annulirostris* mosquitoes were set aside from each trap for processing. Mosquitoes with visual evidence of a recent blood meal were excluded from further analysis. Historically, blood fed mosquitoes have been omitted from processing in order to reduce the likelihood of detecting a virus present solely in the blood meal.

### High-throughput sequencing

A total of 300 mosquitoes were individually washed three times using 750 μl refrigerated phosphate buffered saline, prior to homogenization in 750 μl of cold virus transport medium (in-house formulation; [20]) using the TissueLyserLT (Qiagen, Hilden, Germany) set to 50 KHz for 5 min. For unbiased HTS, we enriched pooled supernatants for virus particles. Aliquots of 50 μl supernatant from each of 25 individual mosquitoes were pooled according to trap for a total of 12 pools. An aliquot of 250 μl pooled material was passed through a 0.45 μM filter (EMD Millipore, Bedford, MA, USA); filtrate was treated with 1.5 μl RNase A (Invitrogen, Carlsbad, CA), 1.8 μl benzonase (EMD Millipore, Billerica, MA, USA) and 2.7 μl 1M MgCl_2_, gently mixed, and left at room temperature for 45 min. Total nucleic acid was extracted from pools using the MagMax Express-96 automated platform (Applied Biosystems, Foster City, CA) with modifications as described by Chidlow et. al. [21]. Nucleic acid concentration and purity was measured on the NanoDrop 1000 spectrophotometer (Thermo Scientific, Wilmington, DE). Total nucleic acid was reverse transcribed using SuperScript III (Invitrogen) and treated with RNAse H (Invitrogen). Double stranded cDNA was prepared using Klenow fragment (3’– 5’ exo-) (New England Biolabs, Beverly, MA). Fragments approximately 200 nt in length were generated by shearing double stranded cDNA on the Focused-Ultrasonicator E210 (Covaris, Woburn, MA). Each library was uniquely barcoded and prepared for sequencing on one lane of the HiSeq 4000 system (Illumina, San Diego, CA) using the Hyper Prep kit (Kapa Biosystems, Boston, MA). Two negative control libraries were also included; the first introduced during sample extraction, and the second during library preparation. A positive control library consisting of the ERCC spike-in was also included.

Sequencing reads generated from HTS processing were demultiplexed, trimmed, and quality filtered using PRINSEQ v0.20.2 [22]. As the *Cx*. *annulirrostris* genome is unavailable, sequences mapping to the *Cx*. *quinquefasciatus* complete genome (NCBI Reference Sequence NZ_AAWU00000000.1) were subtracted using Bowtie2 (v2.0.6, http://bowtie-bio.sourceforge.net) [23] prior to assembly (MEGAHIT v1.0) [24]. Resulting contiguous sequences (contigs) and unique singletons were assessed for sequence similarity to viral reference sequences contained within the non-redundant nucleotide or protein sequence databases of Genbank using MegaBLAST and BLASTx. The sequence of JgV was confirmed using overlapping PCR and bidirectional Sanger sequencing.

### PCR screening for Jogalong virus

Total nucleic acid was prepared from 250 μl of supernatant from the same 300 individual mosquitoes used for HTS. Supernatants were extracted using the MagMax Express-96 platform (Applied Biosystems) as described above and cDNA was prepared from TNA using SuperScript III (Invitrogen). PCR screening primers were designed in the NS5B region using JgV sequences generated from HTS analyses of mosquito pools for the purposes of individual screening (F: CAGGTCCCTATTCTTACACGG; R: TCTGGTAACCGAGGTGTTGC). The identity of all PCR products was confirmed by Sanger sequencing.

### Genome characterization

The hepacivirus polyprotein is co- and post-translationally cleaved using a combination of host proteases (for structural proteins; core, E1, E2, and P7) and viral proteases (for nonstructural proteins; NS2, NS3, NS4A, NS4B, NS5A, and NS5B). We used SignalP 5.0 [25] to identify putative cleavage sites for the structural proteins. To identify the locations of putative cleavage for the nonstructural proteins, we aligned our sequence with other annotated hepacivirus polyproteins and screened for conserved locations.

### Phylogenetics

Protein sequences representing all hepaciviruses including recently described reptilian, fish, and bird hepaciviruses [14–16] were obtained from GenBank, as well as representative pegi- and pestiviruses. All hepacivirus names, sequences and associated hosts are detailed in S1 Table. Two members of the genus *Flavivirus*, represented by Tamana bat virus and yellow fever virus, were included as an outgroup. A conserved region within the NS5 protein [11] was aligned in Geneious 10.2.3 [26] and exported to MEGA6 [27] for phylogenetic analysis. Best-fit model testing was performed within MEGA6 and a maximum likelihood tree was constructed using the Le and Gascuel substitution model [28] with 500 bootstrap repetitions. Newick trees were exported to Figtree (http://tree.bio.ed.ac.uk/software/figtree) for annotation.

### Vertebrate barcoding

Reasoning that an hepacivirus was likely to have a vertebrate host, we performed PCR targeting the cytochrome *b* (cyt *b*) and cytochrome *c* oxidase I (COI) genes found in mitochondrial DNA (mtDNA) [29]. We screened all 50 individual mosquitoes from the Parry’s Creek trap that contained the JgV-positive mosquito. All PCR products were sequenced using the Sanger method. We cloned PCR products using the pGem-T easy vector system (Promega, Madison, WI) to resolve mixed bases that were observed in chromatograms obtained from direct sequencing. Ten colonies were screened for each PCR product.

### Accession numbers

The Jogalong virus sequence has been deposited in GenBank with the accession number MN133813. Illumina sequence data has been deposited in GenBank under BioProject number PRJNA590265.

## Results

### Mosquito collection

The majority of all mosquitoes collected from traps placed across the Kimberley region during March–April 2018 were *Cx*. *annulirostris* (n = 111,019; 58%) [30]. A total of 20,556 *Cx*. *annulirostris* was collected from the six traps located in three sites from across the Kimberley region in the north west of Australia (Table 1, Fig 1); 50 female mosquitoes were randomly selected from each trap for virome analyses.

**Table 1:** *Culex annulirostris* trapped from three sites in the Kimberley region.

Trap	Trap location	Latitude	Longitude	Trap set date	Total trapped
B1	Adjacent to caravan park, Broome	-17.97763161	122.2125935	04/09/18	188*
B2	Cemetery, Broome	-17.957757	122.224449	04/10/18	405*
F1	Rotunda, Fitzroy Crossing	-18.10542047	125.7016503	04/06/18	135
F2	Floodway, Fitzroy Crossing	-18.11554849	125.6773817	04/06/18	170
P1	Jogalong Billabong, Parry’s Creek	-15.59154698	128.261953	03/26/18	1971*
P2	Mangrove, Parry’s Creek	-15.594099	128.288027	03/26/18	17,687*

*Total *Cx*. *annulirostris* extrapolated based on species identification performed on 600 total mosquitoes per trap

### Discovery of Jogalong virus

Sequencing of 12 mosquito pools generated 341 million reads from a single lane of sequencing using the HiSeq 4000 platform (Illumina) (not including controls). A total of 126 million reads were available for assembly following quality filtering and host subtraction. Assembly of reads generated 2.8 million contigs; 32 million unassembled unique singletons remained after assembly. Following BLAST sequence similarity searches, 117,576 (1.7%) sequences (contigs and unique singletons) sourced from mosquito pools shared identity with viral sequences using a minimum MEGABLAST E-value cutoff of 1E-10 or BLASTx cutoff of 1E-3. We identified six contigs (range 201 to 4018 nt; from total of 835 reads) in a single pool from Parry’s Creek that shared low-level identity with hepaciviruses. No hepacivirus-like sequences were observed in negative control samples. We have tentatively named this viral sequence as Jogalong virus (JgV) after the billabong (a seasonal body of water) located near trap P1 at Parry’s Creek.

### Incidence of Jogalong virus

We performed direct PCR on all 300 individual samples collected from the 6 traps distributed across three sites to determine the number of JgV-positive mosquitoes. We found a single JgV-positive mosquito (P1-10) from trap P1 at the Parry’s Creek collection site.

### Virus characterization

**Fig 2:**
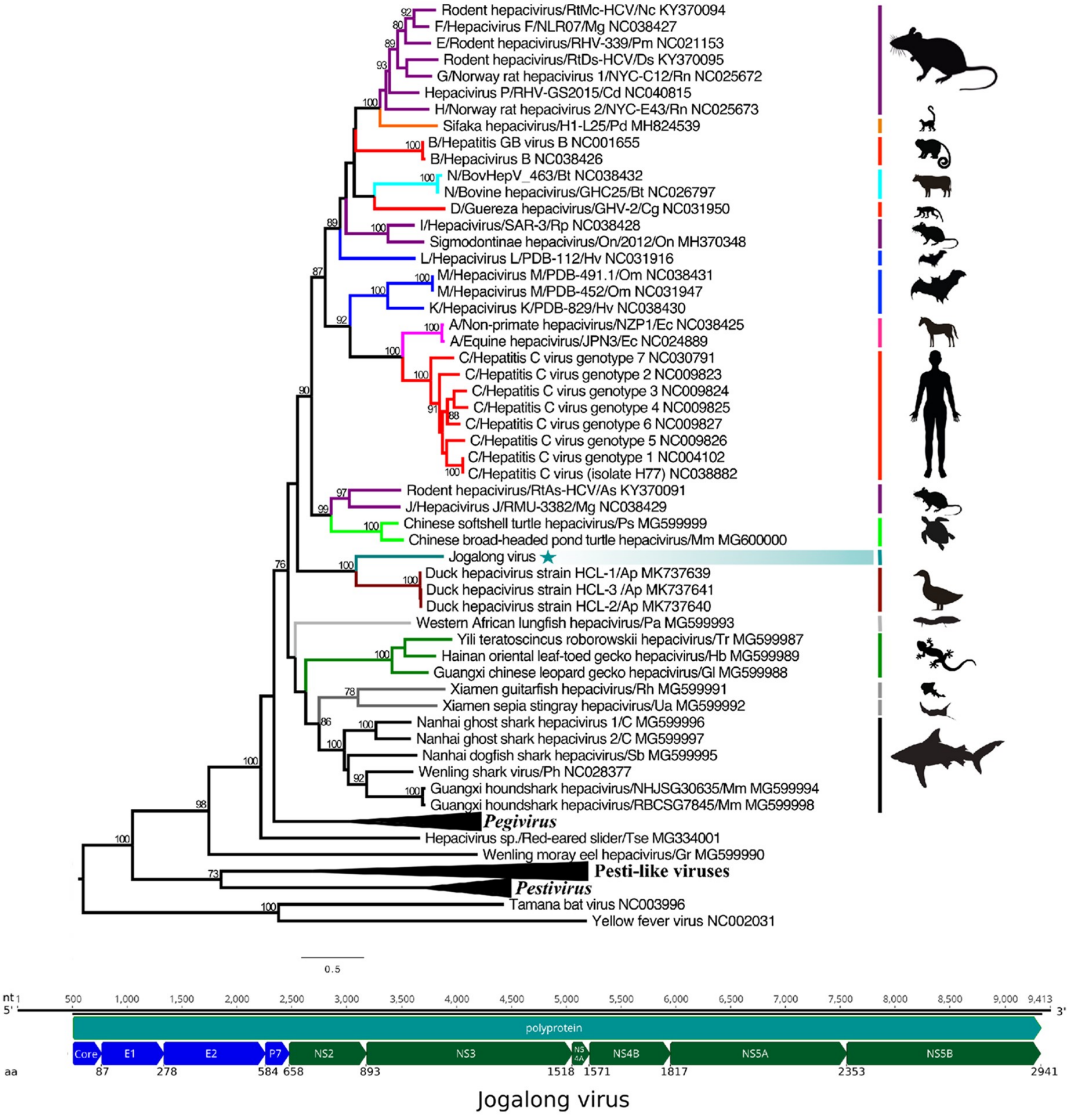
Phylogenetic analysis and putative genome organization of Jogalong virus. The maximum likelihood tree was constructed using a conserved region within the NS5B protein. The scale bar represents substitutions per site and bootstrap values are displayed when greater than 70%. The genera *Pestivirus*, *Pegivirus*, and recently discovered Pesti-like viruses are indicated with a black triangle. Jogalong virus is indicated with a teal star. Hepaciviruses and their associated hosts are indicated by vertical bars. The putative location of the polyprotein (teal arrow) and cleaved structural proteins (blue arrows) and non-structural proteins (dark green arrows) are indicated in the genome map located beneath the tree. Nucleotide (nt) and amino acid (aa) positions can be found above and below the illustration, respectively.

We used PCR on mosquito P1-10 to confirm all contigs that shared identity with hepaciviruses and bridge gaps between sequences obtained from HTS data. PCR primers were designed using assembled HTS data. The complete polyprotein is 8,826 nt (2,941 aa) in length. We identified a hepacivirus-like polyprotein, with putative cellular and viral protease cleavage sites defining 10 co- and post-translationally cleaved proteins (C-E1-E2-p7-NS2-NS3-Ns4a-NS4b-NS5a-NS5b; Table 2, Fig 2). Attempts to identify the complete non-translated genomic regions (NTR) using RACE were unsuccessful. Nonetheless, we were able to confirm 503 nt at the 5’ NTR and 84 nt at the 3’ NTR of the JgV genome using PCR. The presence of a miR-122 binding site could not be located, possibly due to incomplete 5’NTR sequence.

**Table 2:** Hepacivirus protein length and cleave site sequences.

Protein	Core		E1		E2		P7		NS2		NS3		NS4A		NS4B		NS5A		NS5B
Virus	aa	cleavage	aa	cleavage	aa	cleavage	aa	cleavage	aa	cleavage	aa	cleavage	aa	cleavage	aa	cleavage	aa	cleavage	aa
A/Equine hepacivirus/JPN3/Ec	204	GEA|SV	188	VSC|TD	335	AEA|YL	63	AWA|FD	217	RLL|SP	631	TQT|NA	54	EEC|FD	257	QNC|DF	406	ESC|SL	588
B/Hepatitis GB virus B	156	CSG|AR	193	TSG|NP	264	AGL|PL	119*	ASA|FD	208	AIT|AP	620	VNT|SG	55	EEC|AS	248	DDC|GL	411	FSC|SM	590
C/Hepatitis C virus genotype 1	191	ASA|YQ	192	VDA|ET	363	AEA|AL	63	AYA|LD	217	RLL|AP	631	VVT|ST	54	EEC|SQ	261	TPC|SG	448	VCC|SM	591
D/Guereza hepacivirus/GHV-2/Cg	181	GAS|CV	211	VTS|TS	260	AAA|AA	67	AVG|FD	208	SML|NP	625	NDC|SL	60	EEC|SF	248	AQC|DG	882	AKC|AS	591
E/Rodent hepacivirus/RHV-339/Pm	168	ATA|VS	185	AAA|AA	280	AYA|FT	54	AYA|AS	198	KYT|IP	621	FFA|SG	58	EEC|YN	246	DLC|TP	355	HSC|SM	583
F/Hepacivirus F/NLR07/Mg	149	AVT|NC	184	AAA|AS	280	AFA|FT	54	TSA|YS	199	ERT|AP	621	YFA|ST	58	EEC|YQ	246	EDC|SC	479	HEC|SS	585
G/Norway rat hepacivirus 1/NYC-C12/Rn	208	ASA|GI	243	VAA|PV	271	VGA|LE	55	EAY|EG	198	RFT|AP	621	YFA|ET	57	EEC|ST	249	DVC|TS	506	TDC|SW	583
H/Norway rat hepacivirus 2/NYC-E43/Rn	165	AEA|NL	216	SAV|AV	272	SEA|VP	56	RAE|QF	197	QLT|KP	620	YYC|GL	61	EEC|AN	239	EIC|DG	449	SSC|SK	580
I/Hepacivirus/SAR-3/Rp	172	VEP|KP	197	SVA|AP	255	YAQ|PP	53	VEA|FS	204	QLS|SP	622	ELA|SW	56	EEC|AL	251	EPC|TD	385	ETC|TY	586
J/Hepacivirus J/RMU-3382/Mg	163	AVS|HW	185	AEG|LP	287	ANA|LV	44	AQG|GC	236	RLT|AP	625	EEM|TD	58	EEC|GF	258	AEC|AG	555	TSC|NY	603
K/Hepacivirus K/PDB-829/Hv	190	GEA|SY	193	AQA|NP	325	ADA|AL	63	AVG|GP	217	RHC|SP	628	DDT|ST	56	EEC|LS	257	SEC|AF	504	DEC|SA	591
L/Hepacivirus L/PDB-112/Hv	161	AES|VP	203	AAA|MP	265	AWG|WP	59	AQA|AS	212	ERN|AP	629	YSA|GG	57	EEC|MQ	259	AEC|DG	451	ESC|SE	605
M/Hepacivirus M/PDB-491.1/Om	189	VDA|SF	193	SQA|AE	321	ALA|VP	63	VDA|YT	217	RHC|SP	628	TPT|SA	55	EEC|AD	259	RNC|SC	456	SPC|SA	590
N/Bovine hepacivirus/GHC25/Bt	155	VSG|YR	190	VEA|TT	267	ATA|AL	60	VTA|LD	204	APC|AP	624	LDV|WG	54	EEC|WG	250	VPC|GF	390	KEC|SY	579
Hepacivirus P/RHV-GS2015/Cd	153	GLA|FT	186	VAA|PV	270	AEG|AM	56	VLG|AS	199	KRT|AP	622	YFA|ST	58	EEC|SL	245	DFC|SP	395	SDC|SY	580
Sifaka hepacivirus	162	VGA|AF	194	AAA|AP	269	VEA|VP	55	VEA|YT	198	RLT|AP	622	FFT|AW	58	EEC|AL	236	TLC|AS	386	QAL|SQ	576
Wenling shark virus	300	MDS|AP	194	AVA|AP	214	AEA|SV	71	ALG|DD	224	NRC|AP	632	LVA|GL	56	DLS|GA	314	VVM|AD	475	SPM|SH	606
Duck hepacivirus strain HCL-1/Ap	290	ASA|DH	195	GMA|DR	274	AEG|ML	74	VLG|AS	245	QYT|AP	625	NCS|AA	55	EEC|SA	250	YEC|NS	1006	ESC|SF	593
Jogalong virus	87	AVA|FS	191	AQA|GT	306	IEG|AV	74	VAG|ED	235	KLA|AP	625	SAG|LT	52	EEC|AS	247	TNC|TS	536	VCC|GE	588

*p13 is processed into p6 (57aa) and p7 (62aa) in GBV-B; |, location of predicted proteolytic cleavage; further information for each virus can be found in S1 Table.

JgV shares greatest identity with duck hepacivirus across all proteins except for the core and NS4A proteins where there was no apparent identity to any viral sequence (Table 3). Amino acid identity across the structural proteins (E1, E2, and P7) was greatest in the E1 protein (39.4%). Within the non-structural proteins, identity was lowest within the NS2 protein (27.3%) and greatest within the NS3 protein (45.0%). The low sequence identity (or lack thereof) to described viral proteins is supported by phylogenetic analysis of a conserved region within the NS5B protein. Analysis of the partial NS5B protein sequence places JgV outside the diversity of all recognized hepaciviruses on a deeply rooted monophyletic branch shared only with duck hepacivirus (Fig 2). JgV shares a closer phylogenetic relationship to the genus *Hepacivirus* than several recently discovered fish and reptilian hepaciviruses [14, 15]. However, all hepacivirus and hepacivirus-like sequences appear distinct from the clade of viruses belonging to genus *Pegivirus*.

**Table 3:** Jogalong virus protein sequence identity.

Protein	Length (aa)	Closest related viral sequence	Host	Accession	Coverage (%)	E-value	Identity (%)
Core	87	Nil					
E1	191	Duck hepacivirus/HCL-2	*Anas platyrhynchos domesticus* Domestic duck	QDF44087	99%	7.00E-37	39.38%
E2	306	Duck hepacivirus/HCL-2	*Anas platyrhynchos domesticus* Domestic duck	QDF44087	67%	9.00E-25	31.30%
p7	74	Duck hepacivirus/HCL-2	*Anas platyrhynchos domesticus* Domestic duck	QDF44087	79%	4.00E-05	35.59%
NS2	235	Duck hepacivirus/HCL-2	*Anas platyrhynchos domesticus* Domestic duck	QDF44087	98%	8.00E-14	27.31%
NS3	625	Duck hepacivirus/HCL-2	*Anas platyrhynchos domesticus* Domestic duck	QDF44087	100%	1.00E-169	44.99%
NS4A	52	Nil					
NS4B	247	Duck hepacivirus/HCL-2	*Anas platyrhynchos domesticus* Domestic duck	QDF44087	90%	2.00E-31	33.19%
NS5A	536	Duck hepacivirus/HCL-2	*Anas platyrhynchos domesticus* Domestic duck	QDF44087	27%	2.00E-14	36.18%
NS5B	588	Duck hepacivirus/HCL-2	*Anas platyrhynchos domesticus* Domestic duck	QDF44087	96%	3.00E-131	41.51%

JgV has a core protein sequence (87aa) that appears to be much shorter than most other hepaciviruses. Strikingly, the NS5A protein from the closest related viral sequence, duck hepacivirus, was nearly twice as long as the same protein of JgV. All remaining proteins were of similar length to other hepaciviruses (Table 2).

### Blood meal analysis

During mosquito sorting, we excluded mosquitoes with any amount of abdominal swelling consistent with a recent blood meal. However, blood meals can be difficult to visually detect after approximately 60 hours [31]. To investigate whether the JgV-positive mosquito (P1-10) contained a blood meal, we screened all 50 *Cx*. *annulirostris* mosquitoes that were selected from trap P1 at Parry’s Creek. We detected avian mtDNA in a single mosquito, P1-10; the remaining 49 mosquitoes were negative for non-human and non-mosquito mtDNA. The sequence obtained from a single round of COI PCR shared 98.7% nt identity with the tawny frogmouth (*Podargus strigoides*), a native Australian bird species found throughout the country (Table 4). Sequences obtained from direct sequencing of the cyt *b* PCR product indicated co-amplification. Cloning and subsequent sequencing of this amplicon identified *P*. *strigoides* (98.9% nt identity; 5/10 clones) and *Caprimulgus eximius* (golden nightjar; 77.5% nt identity; 5/10 clones). We observed a 9-nt deletion within the *C*. *eximius* sequence that may indicate the co-amplification of nuclear mtDNA paralogs (*numts*). As *numpts* may not be transcribed, we attempted to specifically amplify the COI transcript from P1-10 by DNase-treating total nucleic acid and performing PCR on cDNA [32]. However, we were unable to amplify any product. This may reflect low quality RNA from the digested blood meal. Alternatively, the low identity match to *C*. *eximius* may suggest that there is an avian species in the Kimberley region that is yet to be characterized by mtDNA barcoding techniques.

**Table 4:** Blood meal analysis for mosquito P1-10.

Gene target	PCR	Length of sequence	Proportion of clones	Closest related host sequence	Accession	Common name	Identity (%)
COI	single	305	10/10 (100%)	*Podargus strigoides*	JQ175917	Tawny frogmouth	98.69
Cyt *b*	single	435	5/10 (50%)	*Podargus strigoides*	JQ353838	Tawny frogmouth	98.85
		426	5/10 (50%)	*Caprimulgus eximius*	LT671509	Golden nightjar	77.52

COI, cytochrome *c* oxidase subunit I; cyt *b*, cytochrome b.

## Discussion

We identified nucleic acid sequences of a virus tentatively named Jogalong virus that is related to members of the genus *Hepacivirus*. The sequence was obtained from a single *Cx*. *annulirostris* mosquito collected in the Kimberley region of Western Australia. Large metagenomic surveys of invertebrates are yet to uncover evidence suggesting an invertebrate lineage of hepaciviruses [33]. Thus, all hepaciviruses and hepaci-like viruses identified to date appear to be strictly vertebrate-associated, presumably due to a requirement for the presence of a liver for viral replication. A study of ticks in Australia identified hepacivirus nucleic acid (Collins Beach virus) in an engorged *Ixodes hollocyclus* tick, but that virus is likely associated with the long-nosed bandicoot from which the tick was removed [13]. To our knowledge, JgV represents the second hepacivirus and the first full hepacivirus polyprotein sequence to be discovered from non-human hosts in Australia.

To investigate whether the detection of JgV was associated with a prior blood meal, we performed vertebrate barcoding PCR on all 50 mosquitoes sampled from the Jogalong trap at Parry’s Creek, the site of JgV detection. We detected avian mitochondrial sequences in one of the mosquitoes from this trap; the positive individual corresponded to the JgV-positive mosquito. While more expansive surveillance is required, these data suggest that JgV may have originated from a non-mosquito host. Sequences from two mitochondrial genes closely match the tawny frogmouth; a native, insectivorous bird of the order *Caprimulgiformes* that is found throughout Australia [34]. Investigations of the feeding habits of *Cx*. *annulirostris* mosquitoes indicate that they are generalist feeders exhibiting high host plasticity that include birds [35]. Our results suggest that the blood meal contained JgV nucleic acid. Given the phylogenetic placement of JgV alongside an avian hepacivirus as well as the lack of invertebrate-associated hepaciviruses described to date, we believe it is unlikely that *Cx*. *annulirostris* mosquitoes are the host for this virus.

JgV is a highly divergent hepacivirus and shares only 42% aa identity with its closest relative, duck hepacivirus, across the highly conserved NS5B protein. Duck hepacivirus was recently discovered in China following an investigation of severely diseased ducks. Despite the context of its discovery, the pathogenicity of duck hepacivirus is unclear as the virus was also highly prevalent in healthy ducks [16]. The phylogenetic placement of JgV in a clade shared only with duck hepacivirus offers supporting evidence that JgV is of avian origin. The distant phylogenetic relationship between this potential avian clade (JgV and duck hepacivirus) and all other hepaciviruses is intriguing and suggests a notably different evolutionary history. The vast majority of hepaciviruses identified to date are highly species specific and are thought to have coevolved with their hosts [36]. Thus, a hepacivirus that infects an avian host could be expected to diverge from mammalian, reptilian or fish hepaciviruses. If the blood meal analysis is indicative of the natural host, then JgV may have an avian lineage; however, additional surveillance is required to test this hypothesis.

## Supporting information

S1 Table Hepacivirus names and information. (DOCX)
