# Supplementary material for: Discovery of Jogalong virus, a novel hepacivirus identified in a Culex annulirostris (Skuse) mosquito from the Kimberley region of Western Australia
Source: PLoS One. 2020 Jan 3;15(1):e0227114. doi: 10.1371/journal.pone.0227114 (PMC6941808; doi:10.1371/journal.pone.0227114)
Supplement: S1 Table — (DOCX) [file pone.0227114.s001.docx]

S1 Table. Hepacivirus names and information

| Virus name | *Hepacivirus* species | Accession number | Host scientific name | Host common name |
| --- | --- | --- | --- | --- |
| A/Non-primate hepacivirus/NZP1/Ec | A | NC038425 | *Equus caballus* | Horse |
| A/Equine hepacivirus/JPN3/Ec | A | NC024889 | *Equus caballus* | Horse |
| B/Hepatitis GB virus B | B | NC001655 | NA | New World primate? |
| B/Hepacivirus B | B | NC038426 | NA | New World primate? |
| C/Hepatitis C virus genotype 7 | C | NC030791 | *Homo sapiens* | Human |
| C/Hepatitis C virus genotype 2 | C | NC009823 | *Homo sapiens* | Human |
| C/Hepatitis C virus genotype 3 | C | NC009824 | *Homo sapiens* | Human |
| C/Hepatitis C virus genotype 4 | C | NC009825 | *Homo sapiens* | Human |
| C/Hepatitis C virus genotype 6 | C | NC009827 | *Homo sapiens* | Human |
| C/Hepatitis C virus genotype 5 | C | NC009826 | *Homo sapiens* | Human |
| C/Hepatitis C virus genotype 1 | C | NC004102 | *Homo sapiens* | Human |
| C/Hepatitis C virus (isolate H77) | C | NC038882 | *Homo sapiens* | Human |
| D/Guereza hepacivirus/GHV-2/Cg | D | NC031950 | *Colobus guereza* | Black-and-white colobus |
| E/Rodent hepacivirus/RHV-339/Pm | E | NC021153 | *Peromyscus maniculatus* | Deer mouse |
| F/Hepacivirus F/NLR07/Mg | F | NC038427 | *Myodes glareolus* | European bank vole |
| G/Norway rat hepacivirus 1/NYC-C12/Rn | G | NC025672 | *Rattus norvegicus* | Brown rat |
| H/Norway rat hepacivirus 2/NYC-E43/Rn | H | NC025673 | *Rattus norvegicus* | Brown rat |
| I/Hepacivirus/SAR-3/Rp | I | NC038428 | *Rhabdomys pumilio* | South African four-striped mice |
| J/Hepacivirus J/RMU-3382/Mg | J | NC038429 | *Myodes glareolus* | European bank vole |
| K/Hepacivirus K/PDB-829/Hv | K | NC038430 | *Hipposideros vittatus* | Striped leaf-nosed bat |
| L/Hepacivirus L/PDB-112/Hv | L | NC031916 | *Hipposideros vittatus* | Striped leaf-nosed bat |
| M/Hepacivirus M/PDB-491.1/Om | M | NC038431 | *Otomops martiensseni* | Large-eared free-tailed bat |
| M/Hepacivirus M/PDB-452/Om | M | NC031947 | *Otomops martiensseni* | Large-eared free-tailed bat |
| N/Bovine hepacivirus/GHC25/Bt | N | NC026797 | *Bos taurus* | Cow |
| N/BovHepV_463/Bt | N | NC038432 | *Bos taurus* | Cow |
|  |  |  |  |  |
| Rodent hepacivirus/RtMc-HCV/Nc | Unclassified | KY370094 | *Neodon clarkei* | Clarke's vole |
| Rodent hepacivirus/RtDs-HCV/Ds | Unclassified | KY370095 | *Dipus sagitta* | Northern three-toed jerboa |
| Hepacivirus P/RHV-GS2015/Cd | Unclassified | NC040815 | *Citellus dauricus Brandt* | Ground squirrel |
| Sifaka hepacivirus/H1-L25/Pd | Unclassified | MH824539 | *Propithecus diadema* | Diademed sifaka |
| Sigmodontinae hepacivirus/On/2012/On | Unclassified | MH370348 | *Oligoryzomys nigripes* | Black-footed pygmy rice rat |
| Rodent hepacivirus/RtAs-HCV/As | Unclassified | KY370091 | *Allactaga sibbirica* | Mongolian five-toed jerboa |
| Chinese softshell turtle hepacivirus/Ps | Unclassified | MG599999 | *Pelodiscus sinensis* | Chinese softshell turtle |
| Chinese broad-headed pond turtle hepacivirus/Mm | Unclassified | MG600000 | *Mauremys megalocephala* | Chinese broad-headed pond turtle |
| Duck hepacivirus strain HCL-1/Ap | Unclassified | MK737639 | *Anas platyrhynchos domesticus* | Domestic duck |
| Duck hepacivirus strain HCL-2/Ap | Unclassified | MK737640 | *Anas platyrhynchos domesticus* | Domestic duck |
| Duck hepacivirus strain HCL-3 /Ap | Unclassified | MK737641 | *Anas platyrhynchos domesticus* | Domestic duck |
| Western African lungfish hepacivirus/Pa | Unclassified | MG599993 | *Protopterus annectens* | Western African lungfish |
| Yili teratoscincus roborowskii hepacivirus/Tr | Unclassified | MG599987 | *Teratoscincus roborowskii* | Fog-eyed gecko |
| Hainan oriental leaf-toed gecko hepacivirus/Hb | Unclassified | MG599989 | *Hemidactylus bowringii* | Oriental leaf-toed gecko |
| Guangxi chinese leopard gecko hepacivirus/Gl | Unclassified | MG599988 | *Goniurosaurus luii* | Chinese leopard gecko |
| Xiamen guitarfish hepacivirus/Rh | Unclassified | MG599991 | *Rhinobatos hynnicephalus* | Ringstreaked guitarfish |
| Xiamen sepia stingray hepacivirus/Ua | Unclassified | MG599992 | *Urolophus aurantiacus* | Sepia stingray |
| Nanhai ghost shark hepacivirus 1/C | Unclassified | MG599996 | *Chimaera sp.* | Ghost shark |
| Nanhai ghost shark hepacivirus 2/C | Unclassified | MG599997 | *Chimaera sp.* | Ghost shark |
| Nanhai dogfish shark hepacivirus/Sb | Unclassified | MG599995 | *Squalus brevirostris* | Japanese shortnose spurdog shark |
| Wenling shark virus/Ph | Unclassified | NC028377 | *Proscyllium habereri* | Graceful catshark |
| Guangxi houndshark hepacivirus/NHJSG30635/Mm | Unclassified | MG599994 | *Mustelus manazo* | Starspotted smooth-hound shark |
| Guangxi houndshark hepacivirus/RBCSG7845/Mm | Unclassified | MG599998 | *Mustelus manazo* | Starspotted smooth-hound shark |
